# Genome skimming of dog faecal samples reveals mitogenomes indistinguishable from those of red fox-derived *Uncinaria stenocephala*

**DOI:** 10.1017/S0031182026101565

**Published:** 2026-03

**Authors:** Thomas Stocker, Swaid Abdullah, Ian Scott, Jan Šlapeta

**Affiliations:** 1 Sydney School of Veterinary Science, Faculty of Science, The University of Sydney, Sydney, NSW, Australia; 2 School of Veterinary Science, The University of Queensland, Gatton, QLD, Australia; 3 School of Veterinary Science, Massey University, Palmerston North, New Zealand; 4 Sydney Institute for Infectious Diseases, The University of Sydney, Sydney, NSW, Australia

**Keywords:** canine parasitology, faecal egg isolates, genome skimming, hookworm mitogenome, red fox

## Abstract

The northern hookworm, *Uncinaria stenocephala*, is the primary hookworm infecting dogs in temperate regions, but red foxes (*Vulpes vulpes*) are also frequent hosts. The extent to which fox-derived *U. stenocephala* contributes to canine transmission remains unclear. In this study, we assembled complete mitochondrial genomes (mitogenomes) from two adult *U. stenocephala* worms collected from red fox and two mitogenomes recovered via genome skimming from dog faecal egg isolates. Comparative analysis revealed >99% identity across all *U. stenocephala* mitogenomes with no discernible genetic differences for dog- and fox-derived *U. stenocephala*, supporting their conspecificity. Phylogenetic analysis confirmed paraphyly of the genus *Uncinaria* and clear distinction of *U. stenocephala* from the badger hookworm *U. criniformis*, resolving historical taxonomic ambiguity. We applied a 3% nucleotide divergence threshold to assess species boundaries across hookworm mitogenomes, confirming potential cryptic diversity in *Necator americanus, U. sanguinis* and *A. caninum*. Our findings demonstrate the utility of genome skimming for recovering hookworm mitogenomes from faecal samples and highlight the need for broader mitogenomic characterization across hookworm taxa to refine taxonomy and understand host associations.

## Graphical abstract

**Figure:**
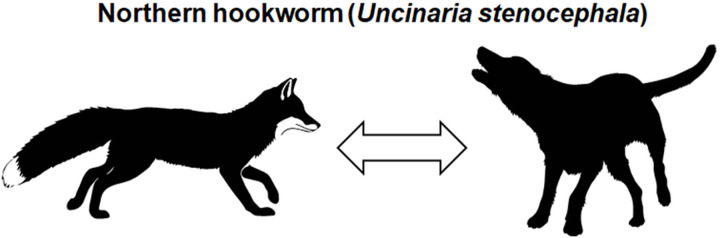

## Introduction

The northern hookworm, *Uncinaria stenocephala*, is the main hookworm species infecting dogs in temperate regions, while *Ancylostoma* species are more common in tropical and subtropical areas (Gibbs, 1961; Beveridge, 2002; Xhaxhiu et al., 2011). Among these, *Ancylostoma caninum* is the most widespread hookworm in dogs from warmer climates and is known to cause anaemia. In contrast, *U. stenocephala* infection usually leads to protein-losing enteropathy rather than anaemia (Walker and Jacobs, 1985).

Hookworm infections are typically treated with benzimidazole-class anthelmintics (Geary et al., 2025). However, resistance to these drugs in *A. caninum* is now a major concern, with evidence suggesting it may be widespread (Geary et al., 2025). Notably, benzimidazole resistance in hookworms may not be limited to *A. caninum* (Leutenegger et al., 2023, 2024; Venkatesan et al., 2023; Abdullah et al., 2025). Recent studies have identified the F167Y mutation in the isotype-1 β-tubulin gene of *U. stenocephala* from Australia, indicating possible resistance in this species too (Abdullah et al., 2025; Chen et al., 2025).

Besides domestic dogs, the European red fox (*Vulpes vulpes*) is a common host of *U. stenocephala* in both Europe and Australia (Coman, 1973; Ryan, 1976; Saeed et al., 2006; Reperant et al., 2007; Bružinskaitė-Schmidhalter et al., 2012; Dybing et al., 2013). The role of fox-derived *U. stenocephala* in dog transmission cycles remains unclear. Three studies have explored this relationship. Two of these studies found, using short molecular markers, that *U. stenocephala* from dogs and foxes are genetically indistinguishable (Górski et al., 2023; Stocker and Šlapeta, 2025). However, an earlier experimental study by Rep and Bos (1979) suggested red foxes may play a limited role in dog infections, as foxes infected with dog-derived *U. stenocephala* produced only low egg burdens compared to dogs.

To better understand the relationship between *U. stenocephala* populations in dogs and foxes, sequencing the mitochondrial genome (mtDNA or mitogenome) is a useful approach. The mitogenome is often the most accessible genome in hookworms, because each hookworm cell contains many mitochondria with multiple mitogenomes compared to singe nuclear genome. Although several hookworm species have had their mitogenomes characterized (Hu et al., 2002; Jex et al., 2009; Gao et al., 2014; Shi et al., 2018; Xie et al., 2019; Tuli et al., 2022), a complete mtDNA sequence for *U. stenocephala* is still unavailable. The mitochondrial *cox*1 gene and the nuclear internal transcribed spacer (ITS) region of the rRNA gene unit are commonly used for molecular barcoding of nematodes (Mejías-Alpízar et al., 2024). While mitochondrial *cox*1 and nuclear ITS sequences from fox-derived *U. stenocephala* have been compared to those from dogs (Górski et al., 2023; Stocker and Šlapeta, 2025), these studies have not examined the full mitochondrial genome.

Sampling adult *U. stenocephala* from dogs is challenging. However, red foxes frequently carry this parasite, making them a practical source (Stocker and Šlapeta, 2025). To overcome the difficulty of obtaining adult worms, a genome skimming approach can be applied to faecal-derived material. Genome skimming involves shallow whole-genome sequencing of complex samples, such as faeces with potentially multiple parasites being present, to identify these parasites, their mitochondrial genomes are assembled and explored for their evolutionary relationships (Papaiakovou et al., 2023).

In this study, we aimed to compare dog- and fox-derived *U. stenocephala* at the molecular level to assess their genetic relatedness. We characterized the complete mitochondrial genome using whole-genome sequencing and applied genome skimming to hookworm stages from dog faeces to expand sampling. We examined both intraspecific and interspecific genetic diversity of the mitogenomes. With access to complete mitogenomes, we investigated the monophyly of the genus *Uncinaria*. Our findings provide baseline insights into the identity of *U. stenocephala* and their conspecificity across red foxes and domestic dogs.

## Material and methods

### Sample isolation and sequencing

Whole-genome sequencing data were obtained from two individual *U. stenocephala* worms (TSF_1.1 and TSF_1.2), collected from a red fox culled in September 2024 in Mardi, New South Wales, Australia (33.2990° S, 151.4084° E) (Stocker and Šlapeta, 2025). Paired-end sequencing reads (FastQ format) were retrieved from GenBank SRA under accession numbers SRR34168471 and SRR34168472 for TSF_1.1 and TSF_1.2, respectively.

Additional DNA samples were obtained from two concentrated hookworm egg samples containing *U. stenocephala* (TS66 from Canberra, Australia; TS78 from Whanganui, New Zealand), and two cultured third-stage larvae (L3) of *A. caninum* (TS87 and TS88 from Queensland, Australia), previously reported by Abdullah et al. (2025). All samples successfully amplified in three PCR assays targeting nuclear ITS2, BZ167 and BZ200 regions, with each assay yielding over 3020 sequencing reads. The *U. stenocephala* samples (TS66 and TS78) did not carry canonical benzimidazole resistance SNPs, while both *A. caninum* samples (TS87 and TS88) showed a high frequency of the F167Y resistance-associated SNP (Abdullah et al., 2025).

Genomic DNA from the faecal egg isolates (TS66 and TS78) and cultured L3 larvae (TS87 and TS88) was submitted to Novogene (Hong Kong) for whole-genome sequencing. Sequencing depth was 3 Gb for TS66 and TS88, and 8 Gb for TS78 and TS87. Sequencing was performed using the Illumina NovaSeq 6000 platform with 150 bp paired-end chemistry.

### Assembly and analysis of *Uncinaria stenocephala* Mitogenomes

Raw FastQ files were assessed using FastQC (http://www.bioinformatics.babraham.ac.uk/projects/fastqc/) and trimmed for low-quality reads and Illumina adapter sequences using Trimmomatic (Bolger et al., 2014). Mitogenomes of *U. stenocephala* (TSF_1.1 and TSF_1.2) and *A. caninum* (TS87) were assembled using GetOrganelle (Jin et al., 2020). Manual annotation was performed by aligning the newly assembled *U. stenocephala* mitogenomes to the reference *A. caninum* mitogenome (NC_012309). Circular genome maps were visualized using Proksee (Grant et al., 2023).

The new mitogenomes were aligned with publicly available hookworm mitogenomes across 12 protein-coding genes. Alignments and phylogenetic analyses were conducted in MEGA12 (Kumar et al., 2024).

Amino acid sequences of the 12 protein-coding genes were translated using the invertebrate mitochondrial genetic code. The alignment was curated using Gblocks v0.91b (Talavera and Castresana, 2007) with default parameters, which retained 3376 of 3777 alignment positions (>99%) across 25 complete hookworm mitochondrial genomes. The mitogenome of *Strongylus vulgaris* was used as the outgroup. The curated alignment was imported into MEGA12 for model selection (Kumar et al., 2024). The best-fit amino acid substitution model was determined to be the Adachi-Hasegawa mitochondrial model (mtREV, Adachi and Hasegawa, 1996) with empirical amino acid frequencies (+*F*), a discrete Gamma distribution with five rate categories to model rate heterogeneity among sites (+*G*), and a proportion of invariant sites (+*I*), resulting in the mtREV + *F* + *G* + *I* model. Phylogenetic trees were constructed using the Maximum Likelihood method implemented in MEGA12. Node support was assessed using adaptive bootstrap resampling (Kumar et al., 2024), with the number of replicates determined automatically by the software with 5% threshold. To assess the robustness of the observed tree topology, particularly the paraphyly of *Uncinaria*, we reconstructed the alignment after excluding the newly assembled *U. stenocephala* mitogenomes. This second alignment, comprising 20 hookworm mitogenomes, was processed using the same Gblocks parameters, retaining 3374 of 3777 residues (>99%). Phylogenetic inference was repeated as described above.

Pairwise nucleotide genetic distances (Kimura 2-parameter, K2P) were calculated in MEGA12 for all sequences. Maximum intraspecific and minimum interspecific distances were extracted and visualized in a scatterplot following the approach of Hebert et al. (2004).

### Mapping of reads to mtDNA, ITS and *β*-tubulin isotype-1 genes

Illumina reads were mapped to the reference mitogenomes of *A. caninum* (TS87) and *U. stenocephala* (TSF_1.1) using BWA-MEM for alignment and SAMtools for file handling (Makino et al., 2024). Reads were also mapped to the nuclear ITS region of *U. stenocephala* (PV848420), β-tubulin isotype-1 gene of *U. stenocephala* (PV133906) and β-tubulin isotype-1 gene of *A. caninum* (DQ459314). Coverage and mapping statistics were calculated using the BBMap pileup.sh command (https://github.com/BioInfoTools/BBMap)

## Results

### Genome skimming reveals high conservation of *Uncinaria stenocephala* ITS sequences

To investigate the molecular identity of *U. stenocephala* from red foxes and domestic dogs, we used the nuclear ITS sequence from an adult worm isolated from a red fox (TSF_1.1; Sanger-derived sequence, PV848420; 818 bp) as a reference. This was compared to low-coverage genome skimming data from two dog faecal samples containing hookworm eggs previously identified as *U. stenocephala* (TS66 from Australia and TS78 from New Zealand) (Abdullah et al., 2025). After quality filtering, the nuclear ITS region showed average coverage of 14.5 × for TS66 and 1032.7 × for TS78, with 85 and 6344 mapped reads, respectively. TS66 had five detectable sequence variants, while TS78 had one. In comparison, the red fox-derived samples (TSF_1.1 and TSF_1.2) had much higher nuclear ITS coverage (9410.1 × and 3809.2 ×, respectively). No sequence variants were detected in TSF_1.1, and only one variant was found in TSF_1.2.

### Characterization of the mitochondrial genome of *Uncinaria stenocephala*

**Figure 1.**
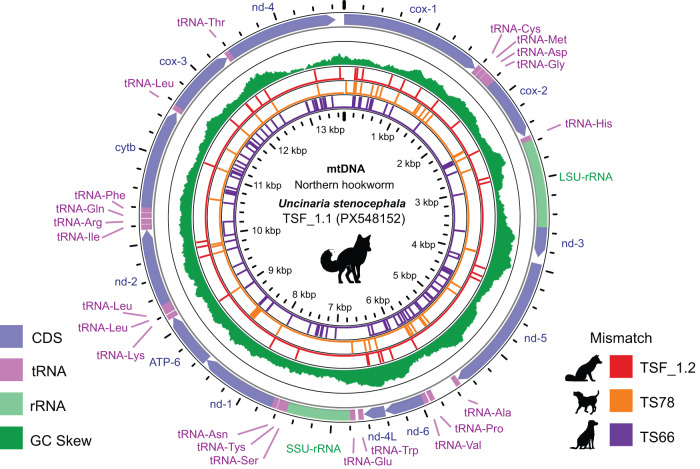
*Uncinaria stenocephala* mitogenome. The circular DNA include 12 protein coding genes (CDS), 2 rRNA genes (rRNA) and 22 tRNA that are depicted on the outermost ring. The inner ring represents the GC content. Following three inner rings with vertical lines represent variable sites compared to the reference (TSF_1.1). The mitogenome of TSF_1.1 and TSF_1.2 were derived from two adult specimens of *U. stenocephala* from a red fox, TS66 and TS78 were derived from canine faecal samples with hookworm egg pools. Figure 1 long description.

Given the high conservation of the nuclear ITS region, we assembled complete circular mitochondrial genomes (mitogenomes) from 2 morphologically verified *U. stenocephala* specimens (TSF_1.1 and TSF_1.2) (Stocker and Šlapeta, 2025). Each mitogenome encoded 12 protein-coding genes, 22 tRNAs, and two rRNAs (Figure 1). Both genomes had a GC content of 22.7%, with thymine being the most frequent nucleotide (49%). Across the 12 coding genes, there were 29 nucleotide differences (99.7% identity). An additional 5 differences were found in non-coding regions. All genes except ND3 contained at least 1 variant, with mitochondrial *cox*1 being the most variable (seven SNPs).

We then mapped low-coverage sequence data from dog-derived samples (TS66 and TS78) to the TSF_1.1 mitogenome to reconstruct dog-derived *U. stenocephala* mitogenomes and assess intraspecific diversity. Mapping of TS78 yielded nearly complete mitogenome coverage, with only 0.5% missing data (38/13 722 bp), all within a hypervariable intergenic region. TS78 had 31,433,349 reads and an average coverage depth of 26.3 × . TS66 had lower coverage (11 336 281 reads; 3.4 × depth), resulting in 88% mitogenome coverage and 12% missing data (1657/13 722 bp) (Table 1).

**Table 1.** Summary statistics for *Uncinaria stenocephala* mitogenomes Table 1 long description.

Sample (host)	Missing nucleotides (%)	Ambiguous nucleotides (%)	Variant sites (%)^a^
TSF_1.1 (red fox)	0 (0)	0 (0)	N/A
TSF_1.2 (red fox)	0 (0)	0 (0)	34 (<1%)
TS66 (dog)^b^	1,657 (12%)	19 (<1%)	21 (<1%)
TS78 (dog)^b^	38 (<1%)	21 (<1%)	19 (<1%)

a Variant sites against a reference (TSF_1.1); N/A not applicable (reference sequence).

b Samples derived from faecal samples with hookworm eggs from an infected dog, representing a population of adults.

For comparison, we also mapped data from two *A. caninum* L3 samples (TS87 and TS88; Abdullah et al., 2025). For *A. caninum*, complete mitogenomes were recovered from TS87 and TS88, with average depths of 442.3 × and 127.2 ×, respectively, from 33 653 543 and 12 515 934 reads.

### *Uncinaria stenocephala* from dogs and red fox represent a single species based on mitogenome identity

**Figure 2.**
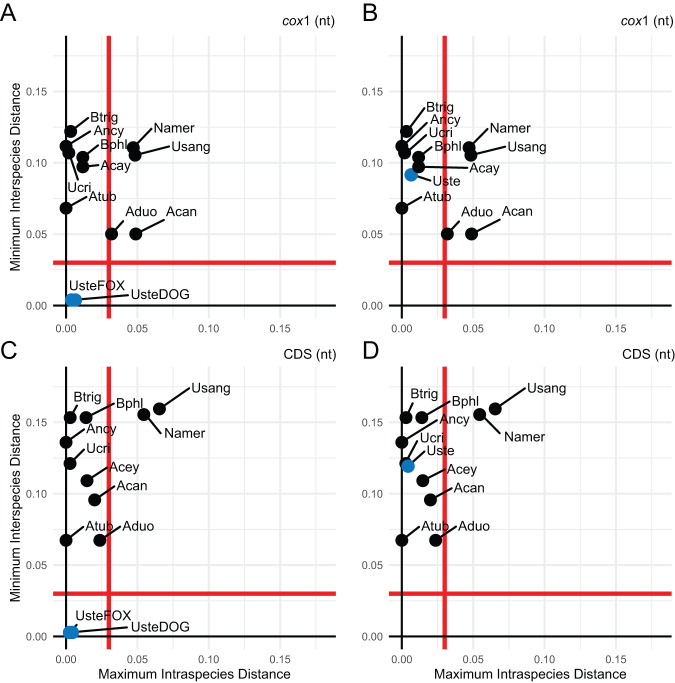
Relationship between intraspecific and interspecific genetic distances among *Uncinaria stenocephala* mitogenomes derived from red foxes and domestic dogs. Maximum intraspecific distances were compared to minimum interspecific congeneric differences using Kimura 2-parameter distances. (A) When analysing only the mitochondrial *cox1* sequence, *U. stenocephala* sequences were divided into two putative host-adapted groups: red fox-derived (UsteFOX) and domestic dog-derived (UsteDOG). (B) In contrast, all *U. stenocephala* sequences were grouped together as a single species (Uste); mitochondrial *cox1* sequence only. (C) When analysing the complete set of mitochondrial protein-coding sequences (CDS), *U. stenocephala* sequences were divided into two putative host-adapted groups: red fox-derived (UsteFOX) and domestic dog-derived (UsteDOG). (D) In contrast, all *U. stenocephala* sequences were grouped together as a single species (Uste) for the CDS-based analysis. Each graph includes a red line representing a 3% divergence threshold, dividing the plot into four quadrants that reflect species categories as defined by Hebert et al. (2004): the top left quadrant indicates species concordant with current taxonomy; the top right suggests probable composite species and candidates for taxonomic split; the bottom left represents species with recent divergence, hybridization, or synonymy; and the bottom right indicates probable specimen misidentification. Species abbreviations used in the figure include: Namer (*Necator americanus*), Usang (*Uncinaria sanguinis*), Ucir (*Uncinaria criniformis*), Acan (*Ancylostoma caninum*), Acey (*Ancylostoma ceylanicum*), Aduo (*Ancylostoma duodenale*), Atub (*Ancylostoma tubaeforme*), Ancy (*Ancylostoma* sp. HL-2021), Bphl (*Bunostomum phlebotomum*) and Btri (*Bunostomum trigonocephalum*). Figure 2 long description.

To assess host-associated divergence, we applied a DNA barcoding approach comparing minimum interspecies distances to maximum intraspecies distances (K2P), using a 3% threshold to define species boundaries (Hebert et al., 2003, 2004). We analysed four *U. stenocephala* mitogenomes (TS66, TS78, TSF_1.1, TSF_1.2) and two *A. caninum* mitogenomes, using both mitochondrial *cox*1 and concatenated coding sequences (CDS) (Figure 2).

When fox- and dog-derived *U. stenocephala* were treated as separate groups, they fell into the bottom-left quadrant of the scatterplot, indicating recent divergence or synonymy (Figure 2A, 2C). When treated as a single species, they shifted to the top-left quadrant, consistent with current taxonomy and so genetically indistinguishable based on mitochondrial *cox*1 and mitochondrial CDS (Figure 2B, 2D). In contrast, *Necator americanus* and *U. sanguinis* appeared in the top-right quadrant, suggesting they may be composite species. *A. duodenale* exceeded the 3% threshold in mitochondrial *cox*1, but not in CDS. *A. caninum* showed a similar pattern, with nearly 5% divergence in mitochondrial *cox*1, driven by the MN215971 mitogenome.

### *Uncinaria stenocephala* from dogs and red fox are monophyletic

**Figure 3.**
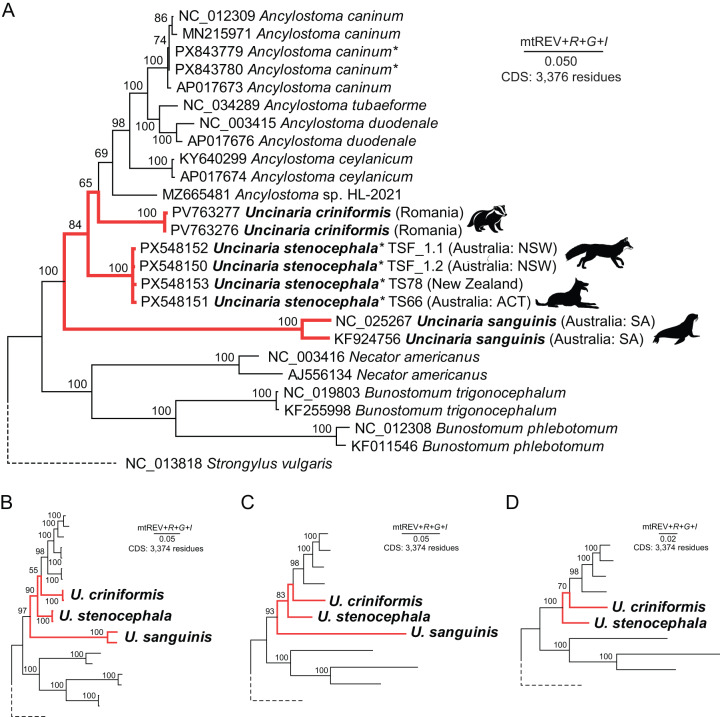
Phylogenetic position of *Uncinaria stenocephala* based on mitochondrial protein-coding gene sequences. Maximum Likelihood trees were inferred using the Adachi and Hasegawa (1996) mitochondrial protein model with empirical amino acid frequencies (+*F*), gamma-distributed rate variation (+*G*), and a proportion of invariant sites (+*I*). Bootstrap support values (adaptively determined) are shown next to the branches. (A) Tree including all available hookworm mitogenomes, including newly assembled *U. stenocephala* (*n* = 4) and *A. caninum* (*n* = 2) sequences. Newly generated sequences are marked with an asterisk (*). Pictogram of host animal is next to *Uncinaria* spp. (badger, fox, dog, sea lion) (B) Tree excluding all newly generated mitogenomes from genome skimming. (C) Tree with only a single representative per hookworm species. (D) Tree with *U. sanguinis* removed and a single representative retained for each remaining species. In panels (B)–(D), only nodes corresponding to *Uncinaria* species are labelled. Figure 3 long description.

Using the newly assembled *U. stenocephala* mitochondrial genomes, we constructed a phylogenetic tree based on amino acid sequences from all 12 protein-coding genes. The maximum likelihood tree was inferred using the mtREV + *F* + *G* + *I* model. The genus *Uncinaria* appeared paraphyletic, with *U. sanguinis* (from pinnipeds) forming a distinct lineage separate from *U. stenocephala, U. criniformis* (from badgers) and all *Ancylostoma* spp. sequences (Figure 3). Bootstrap support for each individual *Uncinaria* species (*U. stenocephala, U. criniformis, U. sanguinis*) was absolute (100%) (Figure 3A). Bootstrap support for the nodes leading to *Uncinaria* species and the unnamed *Ancylostoma* sp. HL-2021 (MZ665481) was lower (65–84%) than in other parts of the tree (Figure 3A). This paraphyly persisted regardless of which sequences or species were excluded with variable bootstrap support for the nodes that led to paraphyletic *Uncinaria* clades. To further investigate, we removed *Ancylostoma* sp. HL-2021 and retained a maximum of two representatives per species. The tree was recalculated using the same model, yet *Uncinaria* species remained paraphyletic (Figure 3B). We then retained only a single representative per species (Figure 3C), and finally, we removed *U. sanguinis* entirely (Figure 3D). The paraphyly of *U. stenocephala* and *U. criniformis* and tree topology remained unchanged. The apparent paraphyly of *Uncinaria* remains unresolved, because the weak nodal support (<70% bootstrap) suggests that inclusion of additional *Uncinaria* spp. mitogenomes may improve tree resolution (Ilík et al., 2024; Deak and Šlapeta, 2025).

### Limited coverage of *β*-tubulin isotype-1 gene from genome skimming

Genome skimming of the *U. stenocephala* sample TS78 yielded only 22 reads mapping to the β-tubulin isotype-1 (tubb-1) gene, out of 62 866 698 total reads. These reads did not cover key codons (134, 167, 198, 200) associated with benzimidazole resistance. No reads mapped to tubb-1 in the TS66 sample.

In contrast, *A. caninum* L3 samples (TS87 and TS88) had better coverage of tubb-1 (7.7 × and 7.9 ×), but the gene was still only partially covered, with no reads spanning the benzimidazole resistance-associated codons.

## Discussion

The mitogenomes of *U. stenocephala* recovered from red foxes were highly similar to those assembled from dog faecal egg isolates via genome skimming, with pairwise identity exceeding 99%. The genetic distance between fox- and dog-derived *U. stenocephala* was lower than that observed among *A. caninum* isolates, including three from Australian dogs. These findings support earlier studies based on nuclear ITS and partial mitochondrial *cox*1 sequences, which found no clear genetic distinction between *U. stenocephala* from dogs and foxes (Górski et al., 2023; Stocker and Šlapeta, 2025). Genome skimming proved to be a practical and effective method for recovering complete or near-complete mitogenomes from faecal samples, especially when adult nematodes are inaccessible due to host status or sampling constraints (Papaiakovou et al., 2023). Even shallow Illumina sequencing provided sufficient coverage for mitogenome reconstruction and the nuclear multicopy rDNA unit; however, it failed to yield adequate coverage for single-copy nuclear genes such as β-tubulin isotype-1, for which deep amplicon sequencing remains the more appropriate approach when considering benzimidazole resistance alleles (Venkatesan et al., 2023; Abdullah et al., 2025; Chen et al., 2025).

In contrast to the genetic evidence, earlier experimental work by Rep and Bos (1979) suggested that *U. stenocephala* strains in dogs and foxes may be host-adapted, with limited cross-infectivity. Their study showed that dog-derived *U. stenocephala* produced patent infections in foxes but with markedly reduced egg output, only 7% compared to dog-to-dog transmission, and less than 1% when fox-derived worms were used to infect dogs (Rep and Bos, 1979). However, faecal egg counts are influenced by multiple factors, including worm burden, sex ratio and daily variation in egg shedding (Watkins and Harvey, 1942). Additionally, the experimental infections used vastly different larval doses (25 000 vs 1000), and some dogs expelled larvae orally due to high infection pressure. The immune status of the hosts was not well characterized, and prior exposure may have influenced larval establishment (Ferguson et al., 2023). While our mitogenomic data support conspecificity of *U. stenocephala* from foxes and dogs, we cannot rule out phenotypic variation or host-specific adaptation. Further studies using variable nuclear markers across broader geographic regions are needed to clarify the contribution of fox-derived *U. stenocephala* to dog infections.

Molecular markers such as mitogenomes and nuclear ITS regions are increasingly recognized as complementary characteristics to morphology in hookworm systematics (Hu et al., 2002; Jex et al., 2009; Gao et al., 2014; Shi et al., 2018; Xie et al., 2019). For example, the distinction between *A. braziliense* and *A. ceylanicum* was only resolved through DNA sequence analysis, particularly nuclear ITS region, which clarified their taxonomic status and ended longstanding speculation about their synonymy (Traub et al., 2008; Ngui et al., 2013; Traub, 2013). Despite the public health relevance of *A. braziliense*, no complete mitogenome is currently available for this species. In fact, only a small number of morphologically described hookworm species have been characterized at the nucleotide level, and even fewer have complete mitogenomes (Ilík et al., 2024).

In this study, we applied an arbitrary 3% threshold to assess nucleotide variability across available hookworm mitogenomes. When considering all protein-coding sequences, all but two species were consistent with current taxonomy. The exceptions were *Necator americanus* and *Uncinaria sanguinis*. In the case of *U. sanguinis*, a hookworm of pinnipeds, it is the only species within this group with published mitogenomes, despite the existence of numerous loosely host-associated *Uncinaria* species in pinnipeds worldwide, often characterized using partial mitochondrial *cox*1 and nuclear ITS sequences (Nadler et al., 2013; Haynes et al., 2014). This group likely harbours cryptic diversity, and genome skimming combined with mitogenomics directly from faecal samples collected antemortem could provide new insights into the taxonomy and evolutionary relationships within the genus. For *N. americanus*, mitogenomic variability was first noted by Hu et al. (2003) when comparing samples from Togo and China, but no follow-up studies have explored whether these differences reflect biologically distinct populations. Interestingly, when we restricted our analysis to the mitochondrial *cox*1 gene alone, *A. caninum*, previously grouped with well-defined taxa, shifted into the quadrant suggesting potential cryptic species. This divergence was driven by the mitochondrial *cox*1 sequence from MN215971 (Xie et al., 2019), derived from a stray dog in a shelter in Southwest China. Further sampling is needed to determine whether this divergence reflects local variation, a technical artefact, or genuine cryptic diversity, as the remainder of the mitogenome does not show similar divergence.

## Conclusions

The characterization of the *Uncinaria stenocephala* mitogenome and its clear genetic distinction from the badger hookworm *U. criniformis* resolves longstanding speculation that these species may be synonymous (Ransom, 1924; Deak and Šlapeta, 2025). Although morphologically similar, the genetic distance between their mitogenomes was greater than previously assumed, challenging historical interpretations based solely on morphology (Looss, 1905; Cameron, 1924; Ransom, 1924; Baylis, 1933; Wolfgang, 1956).

This study contributes to resolving hookworm taxonomy and highlights the utility of mitogenomics in clarifying species boundaries. However, the genus *Uncinaria* remains polyphyletic. Further mitogenomic characterization is needed across additional *Uncinaria* species and related genera, including *Agriostomum, Arthrocephalus, Arthrosoma, Galoncus, Globocephalus, Hypodontus, Placoconus* and *Tetragomphius* (Ilík et al., 2024; Deak and Šlapeta, 2025). Expanding genomic datasets across these taxa will be essential for resolving phylogenetic relationships and refining the taxonomy of hookworms and related nematodes.

## Data Availability

The raw and intermediate data for all samples are available at LabArchives (https://dx.doi.org/10.25833/j9wf-9s30). Raw FastQ sequence data were deposited at SRA NCBI BioProject: PRJNA1347473 (https://www.ncbi.nlm.nih.gov/bioproject/PRJNA1347473). Mitogenome sequence data were deposited in GenBank under the following accession numbers: PX548150-PX548153 and PX843779-PX843780.

## Figure 1 Long description

Insufficient visual information to describe this element accurately.

Navigate back to Figure 1.

## Figure 2 Long description

The image A showing a scatter plot labeled “cox1 (nt)”. The horizontal axis label is “Maximum Intraspecies Distance” with tick labels 0.00, 0.05, 0.10, 0.15. The vertical axis label is “Minimum Interspecies Distance” with tick labels 0.00, 0.05, 0.10, 0.15. A vertical reference line is drawn at 0.03 on the horizontal axis and a horizontal reference line is drawn at 0.03 on the vertical axis. Labeled points include: Btrig, Ancy, Bphl, Acay, Ucri, Atub, Namer, Usang, Aduo, Acan, UsteFOX, UsteDOG. UsteFOX is plotted near (0.00, 0.00). UsteDOG is plotted near (0.03, 0.00). Several labeled points are plotted with minimum interspecies distance above 0.05 and maximum intraspecies distance between 0.00 and 0.06. The image B showing a scatter plot labeled “cox1 (nt)”. The horizontal axis label is “Maximum Intraspecies Distance” with tick labels 0.00, 0.05, 0.10, 0.15. The vertical axis label is “Minimum Interspecies Distance” with tick labels 0.00, 0.05, 0.10, 0.15. A vertical reference line is drawn at 0.03 on the horizontal axis and a horizontal reference line is drawn at 0.03 on the vertical axis. Labeled points include: Btrig, Ancy, Ucri, Bphl, Uste, Atub, Acay, Namer, Usang, Aduo, Acan. The point labeled Uste is plotted near (0.00, 0.09) and is marked with a different fill style than the other points. Several labeled points are plotted with minimum interspecies distance above 0.05 and maximum intraspecies distance between 0.00 and 0.06. The image C showing a scatter plot labeled “CDS (nt)”. The horizontal axis label is “Maximum Intraspecies Distance” with tick labels 0.00, 0.05, 0.10, 0.15. The vertical axis label is “Minimum Interspecies Distance” with tick labels 0.00, 0.05, 0.10, 0.15. A vertical reference line is drawn at 0.03 on the horizontal axis and a horizontal reference line is drawn at 0.03 on the vertical axis. Labeled points include: Btrig, Bphl, Ancy, Ucri, Acey, Acan, Atub, Aduo, Usang, Namer, UsteFOX, UsteDOG. UsteFOX is plotted near (0.00, 0.00). UsteDOG is plotted near (0.03, 0.00). Several labeled points are plotted with minimum interspecies distance above 0.05 and maximum intraspecies distance between 0.00 and 0.08. The image D showing a scatter plot labeled “CDS (nt)”. The horizontal axis label is “Maximum Intraspecies Distance” with tick labels 0.00, 0.05, 0.10, 0.15. The vertical axis label is “Minimum Interspecies Distance” with tick labels 0.00, 0.05, 0.10, 0.15. A vertical reference line is drawn at 0.03 on the horizontal axis and a horizontal reference line is drawn at 0.03 on the vertical axis. Labeled points include: Btrig, Bphl, Ancy, Ucri, Uste, Acey, Acan, Atub, Aduo, Usang, Namer. The point labeled Uste is plotted near (0.00, 0.12) and is marked with a different fill style than the other points. Several labeled points are plotted with minimum interspecies distance above 0.05 and maximum intraspecies distance between 0.00 and 0.08. Across the four scatter plots, the vertical and horizontal reference lines at 0.03 divide each plot into four regions. In all four plots, multiple labeled points appear above the horizontal 0.03 line. In images A and C, UsteFOX is near (0.00, 0.00) and UsteDOG is near (0.03, 0.00). In images B and D, Uste appears near (0.00, 0.09) in image B and near (0.00, 0.12) in image D and is shown with a different fill style than the other points.

Navigate back to Figure 2.

## Figure 3 Long description

The image contains four phylogenetic trees labeled A, B, C and D, illustrating the relationships among various hookworm species based on mitochondrial protein-coding gene sequences. In tree A, the maximum likelihood tree includes all available hookworm mitogenomes, with newly assembled sequences marked by an asterisk. The tree is rooted with Strongylus vulgaris and shows bootstrap support values next to the branches. The tree uses the mtREV plus F plus G plus I model, with a scale bar indicating 0.05 substitutions per site. The tree highlights Uncinaria criniformis and Uncinaria stenocephala in red, with pictograms of host animals such as badger, fox, dog and sea lion next to the species names. Tree B excludes newly generated mitogenomes, showing only Uncinaria criniformis, Uncinaria stenocephala and Uncinaria sanguinis, with bootstrap values indicated. Tree C retains a single representative per hookworm species, showing similar relationships with bootstrap values. Tree D removes Uncinaria sanguinis entirely, retaining a single representative for each remaining species, with the tree topology remaining unchanged. Each tree uses the same model and scale, with the number of coding sequences and residues noted.

Navigate back to Figure 3.

## Table 1 Long description

The table summarizes mitogenome assembly quality and sequence differences for four Uncinaria stenocephala samples from red fox and dog hosts. For each sample it reports the count and percent of missing nucleotides, the count and percent of ambiguous nucleotides, and the number and percent of variant sites relative to a designated reference sequence. The reference sample TSF_1.1 (red fox) has 0 missing and 0 ambiguous nucleotides, and variant sites are not applicable because it is the reference. TSF_1.2 (red fox) also has 0 missing and 0 ambiguous nucleotides, with 34 variant sites representing less than 1% of positions. Among dog-derived samples, TS66 has the highest missing data at 1,657 nucleotides (12%) and 19 ambiguous nucleotides (under 1%), with 21 variant sites (under 1%). TS78 has low missing data at 38 nucleotides (under 1%) and 21 ambiguous nucleotides (under 1%), with 19 variant sites (under 1%). Overall, ambiguity is consistently low across all samples, while missing data varies most strongly in TS66; variant-site percentages are uniformly under 1% for all non-reference sequences. Variant counts are relative to the chosen reference, and the dog samples are derived from fecal material, which may affect completeness compared with the fox assemblies.

Navigate back to Table 1.

## References

[ref1] Abdullah S, Stocker T, Kang H, Scott I, Hayward D, Jaensch S, Ward MP, Jones MK, Kotze AC and Šlapeta J (2025) Widespread occurrence of benzimidazole resistance single nucleotide polymorphisms in the canine hookworm, *Ancylostoma caninum*, in Australia. *International Journal for Parasitology* 55(3-4), 173–182. 10.1016/j.ijpara.2024.12.001 39716589

[ref2] Adachi J and Hasegawa M (1996) Model of amino acid substitution in proteins encoded by mitochondrial DNA. *Journal of Molecular Evolution* 42(4), 459–468. 10.1007/BF02498640 8642615

[ref3] Baylis HA (1933) A new species of the nematode genus *Uncinaria* from a sea-lion, with some observations on related species. *Parasitology* 25(3), 308–316. 10.1017/S0031182000019508

[ref4] Beveridge I (2002) Australian hookworms (Ancylostomatoidea): A review of the species present, their distributions and biogeographical origins. *Parassitologia* 44(1-2), 83–88. 12404813

[ref5] Bolger AM, Lohse M and Usadel B (2014) Trimmomatic: a flexible trimmer for Illumina sequence data. *Bioinformatics* 30(15), 2114–2120. 10.1093/bioinformatics/btu170 24695404 PMC4103590

[ref6] Bružinskaitė-Schmidhalter R, Šarkūnas M, Malakauskas A, Mathis A, Torgerson PR and Deplazes P (2012) Helminths of red foxes (*Vulpes vulpes*) and raccoon dogs (*Nyctereutes procyonoides*) in Lithuania. *Parasitology* 139(1), 120–127. 10.1017/S0031182011001715 21996514

[ref7] Cameron TWM (1924) *Dochmoides*: a new geuns for the hookworm “*Uncinaria*” *stenocephala* Railliet. *Journal of Helminthology* 2(1), 46–50. 10.1017/S0022149X00002996

[ref8] Chen Y-J, Li V, Suwandy M, Mitrea IB, Hayward D, Jaensch S, Francis EK and Šlapeta J (2025) Ambient temperature storage in DESS supports molecular studies of benzimidazole resistance from canine hookworm eggs. *International Journal for Parasitology* in press. 10.1016/j.ijpara.2025.10.005. 41135798

[ref9] Coman BJ (1973) Helminth Parasites Of the Fox (*Vulpes vulpes*) In Victoria. *Australian Veterinary Journal* 49(8), 378–384. 10.1111/j.1751-0813.1973.tb09345.x 4795938

[ref10] Deak G and Šlapeta J (2025) Molecular characterization and reference mitogenome of the hookworm *Uncinaria criniformis* (Goeze, 1782) from the Eurasian badger. *Parasitology* 152 (10), 1070–1074. 10.1017/S0031182025100760 40826957 PMC12644965

[ref11] Dybing NA, Fleming PA and Adams PJ (2013) Environmental conditions predict helminth prevalence in red foxes in Western Australia. *International Journal for Parasitology: Parasites and Wildlife* 2, 165–172. 10.1016/j.ijppaw.2013.04.004 24533331 PMC3862530

[ref12] Ferguson AA, Inclan-Rico JM, Lu D, Bobardt SD, Hung L, Gouil Q, Baker L, Ritchie ME, Jex AR, Schwarz EM, Rossi HL, Nair MG, Dillman AR and Herbert DR (2023) Hookworms dynamically respond to loss of Type 2 immune pressure. *PLOS Pathogens* 19(12), e1011797. 10.1371/journal.ppat.1011797 38079450 PMC10735188

[ref13] Gao JF, Zhao Q, Liu GH, Zhang Y, Zhang Y, Wang WT, Chang QC, Wang CR and Zhu XQ (2014) Comparative analyses of the complete mitochondrial genomes of the two ruminant hookworms *Bunostomum trigonocephalum* and *Bunostomum phlebotomum*. *Gene* 541(2), 92–100. 10.1016/j.gene.2014.03.017 24625354

[ref14] Geary TG, Drake J, Gilleard JS, Chelladurai JRJJ, Jimenez Castro PD, Kaplan RM, Marsh AE, Reinemeyer CR and Verocai GG (2025) Multiple anthelmintic drug resistance in the canine hookworm *Ancylostoma caninum*: AAVP position paper and research needs. *Veterinary Parasitology* 338, 110536. 10.1016/j.vetpar.2025.110536 40596793

[ref15] Gibbs HC (1961) Studies on the life cycle and developmental morphology of *Dochmoides stenocephala* (Railliet 1884) (Ancylostomidae: Nematoda). *Canadian Journal of Zoology* 39(3), 325–348. 10.1139/z61-037

[ref16] Górski P, Długosz E, Bartosik J, Bąska P, Łojek J, Zygner W, Karabowicz J and Wiśniewski M (2023) Morphological and molecular comparison of nematodes from the family Ancylostomatidae isolated from selected species of carnivorous mammals in central Poland. *Medycyna Weterynaryjna* 79(5), 223–226. 10.21521/mw.6766

[ref17] Grant JR, Enns E, Marinier E, Mandal A, Herman EK, Chen CY, Graham M, Van Domselaar G and Stothard P (2023) Proksee: In-depth characterization and visualization of bacterial genomes. *Nucleic Acids Research* 51(W1), W484–W492. 10.1093/nar/gkad326 37140037 PMC10320063

[ref18] Haynes BT, Marcus AD, Higgins DP, Gongora J, Gray R and Šlapeta J (2014) Unexpected absence of genetic separation of a highly diverse population of hookworms from geographically isolated hosts. *Infection Genetics & Evolution* 28, 192–200. 10.1016/j.meegid.2014.09.022 25262830

[ref19] Hebert PD, Cywinska A, Ball SL and deWaard JR (2003) Biological identifications through DNA barcodes. *Proceedings of the Royal Society of London Series B Biological Sciences* 270(1512), 313–321. 10.1098/rspb.2002.2218 PMC1691236 12614582

[ref20] Hebert PD, Stoeckle MY, Zemlak TS and Francis CM (2004) Identification of birds through DNA barcodes. *PLoS Biology* 2(10), e312. 10.1371/journal.pbio.0020312 15455034 PMC518999

[ref21] Hu M, Chilton NB and Gasser RB (2002) The mitochondrial genomes of the human hookworms, *Ancylostoma duodenale* and *Necator americanus* (Nematoda: Secernentea). *International Journal for Parasitology* 32(2), 145–158. 10.1016/s0020-7519(01)00316-2 11812491

[ref22] Hu M, Chilton NB, YG AE-O and Gasser RB (2003) Comparative analysis of mitochondrial genome data for *Necator americanus* from two endemic regions reveals substantial genetic variation. *International Journal for Parasitology* 33(9), 955–963. 10.1016/s0020-7519(03)00129-2 12906879

[ref23] Ilík V, Schwarz EM, Nosková E and Pafčo B (2024) Hookworm genomics: Dusk or dawn? *Trends in Parasitology* 40(6), 452–465. 10.1016/j.pt.2024.04.003 38677925

[ref24] Jex AR, Waeschenbach A, Hu M, van Wyk JA, Beveridge I, Littlewood DT and Gasser RB (2009) The mitochondrial genomes of *Ancylostoma caninum* and *Bunostomum phlebotomum* -two hookworms of animal health and zoonotic importance. *BMC Genomics* 10, 79. 10.1186/1471-2164-10-79 19210793 PMC2656527

[ref25] Jin JJ, Yu WB, Yang JB, Song Y, dePamphilis CW, Yi TS and Li DZ (2020) GetOrganelle: a fast and versatile toolkit for accurate de novo assembly of organelle genomes. *Genome Biology* 21(1), 241. 10.1186/s13059-020-02154-5 32912315 PMC7488116

[ref26] Kumar S, Stecher G, Suleski M, Sanderford M, Sharma S and Tamura K (2024) MEGA12: Molecular evolutionary genetic analysis version 12 for adaptive and green computing. *Molecular Biology and Evolution* 41(12). 10.1093/molbev/msae263 PMC11683415 39708372

[ref27] Leutenegger CM, Evason MD, Willcox JL, Rochani H, Richmond HL, Meeks C, Lozoya CE, Tereski J, Loo S, Mitchell K, Andrews J and Savard C (2024) Benzimidazole F167Y polymorphism in the canine hookworm, *Ancylostoma caninum*: Widespread geographic, seasonal, age, and breed distribution in United States and Canada dogs. *International Journal for Parasitology: Drugs and Drug Resistance* 24, 100520. 10.1016/j.ijpddr.2024.100520 38237210 PMC10825515

[ref28] Leutenegger CM, Lozoya CE, Tereski J, Savard C, Ogeer J and Lallier R (2023) Emergence of *Ancylostoma caninum* parasites with the benzimidazole resistance F167Y polymorphism in the US dog population. *International Journal for Parasitology: Drugs and Drug Resistance* 21, 131–140. 10.1016/j.ijpddr.2023.01.001 36958067 PMC10068012

[ref29] Looss A (1905) *The Anatomy and Life History of Ancylostoma Duodenale Dubini*. Cairo, Egypt: Records of the School of Medicine.

[ref30] Makino J, Ebisuzaki T, Himeno R and Hayashizaki Y (2024) Fast and accurate short-read alignment with hybrid hash-tree data structure. *Genomics & Informatics* 22(1), 19. 10.1186/s44342-024-00012-5 39472988 PMC11520436

[ref31] Mejías-Alpízar MJ, Porras-Silesky C, Rodríguez EJ, Quesada J, Alfaro-Segura MP, Robleto-Quesada J, Gutiérrez R and Rojas A (2024) Mitochondrial and ribosomal markers in the identification of nematodes of clinical and veterinary importance. *Parasites and Vectors* 17(1), 77. 10.1186/s13071-023-06113-4 38378676 PMC10880205

[ref32] Nadler SA, Lyons ET, Pagan C, Hyman D, Lewis EE, Beckmen K, Bell CM, Castinel A, Delong RL, Duignan PJ, Farinpour C, Huntington KB, Kuiken T, Morgades D, Naem S, Norman R, Parker C, Ramos P, Spraker TR and Beron-Vera B (2013) Molecular systematics of pinniped hookworms (Nematoda: *Uncinaria*): Species delimitation, host associations and host-induced morphometric variation. *International Journal for Parasitology* 43(14), 1119–1132. 10.1016/j.ijpara.2013.08.006 24162075

[ref33] Ngui R, Mahdy MA, Chua KH, Traub R and Lim YA (2013) Genetic characterization of the partial mitochondrial cytochrome oxidase c subunit I (cox 1) gene of the zoonotic parasitic nematode, *Ancylostoma ceylanicum* from humans, dogs and cats. *Acta Tropica* 128(1), 154–157. 10.1016/j.actatropica.2013.06.003 23774318

[ref34] Papaiakovou M, Fraija-Fernandez N, James K, Briscoe AG, Hall A, Jenkins TP, Dunn J, Levecke B, Mekonnen Z, Cools P, Doyle SR, Cantacessi C and Littlewood DTJ (2023) Evaluation of genome skimming to detect and characterise human and livestock helminths. *International Journal for Parasitology* 53(2), 69–79. 10.1016/j.ijpara.2022.12.002 36641060

[ref35] Ransom BH (1924) Hookworms of the genus *Uncinaria* of the dog, fox, and badger. *Proceedings of the United States National Museum* 65(2533), 1–5. 10.5479/si.00963801.65-2533.1

[ref36] Rep BH and Bos R (1979) Enige epidemiologische aspekten van *Uncinaria stenocephala* infecties in Nederland. *Tijdschr Diergeneeskd* 104, 747–758. 573511

[ref37] Reperant LA, Hegglin D, Fischer C, Kohler L, Weber JM and Deplazes P (2007) Influence of urbanization on the epidemiology of intestinal helminths of the red fox (*Vulpes vulpes*) in Geneva, Switzerland. *Parasitology Research* 101(3), 605–611. 10.1007/s00436-007-0520-0 17393184

[ref38] Ryan GE (1976) Helminth parasites of the fox (*Vulpes vulpes* ) in New South Wales. *Australian Veterinary Journal* 52(3), 126–131. 10.1111/j.1751-0813.1976.tb05445.x 985241

[ref39] Saeed I, Maddox-Hyttel C, Monrad J and Kapel CM (2006) Helminths of red foxes (*Vulpes vulpes*) in Denmark. *Veterinary Parasitology* 139(1-3), 168–179. 10.1016/j.vetpar.2006.02.015 16580775

[ref40] Shi X, Wang M, Abdullahi AY, Fu Y, Yang F, Yu X, Pan W, Yan X, Hang J, Zhang P and Li G (2018) Comparative analysis of *Ancylostoma ceylanicum* mitochondrial genome with other *Ancylostoma* species. *Infection Genetics & Evolution* 62, 40–45. 10.1016/j.meegid.2018.04.012 29660556

[ref41] Stocker T and Šlapeta J (2025) Characterisation of β-tubulin isotypes in *Uncinaria stenocephala* and implications for benzimidazole resistance in hookworms. *Veterinary Parasitology* 339, 110569. 10.1016/j.vetpar.2025.110569 40774129

[ref42] Talavera G and Castresana J (2007) Improvement of phylogenies after removing divergent and ambiguously aligned blocks from protein sequence alignments. *Systematic Biology* 56(4), 564–577. 10.1080/10635150701472164 17654362

[ref43] Traub RJ (2013) *Ancylostoma ceylanicum*, a re-emerging but neglected parasitic zoonosis. *International Journal for Parasitology* 43(12-13), 1009–1015. 10.1016/j.ijpara.2013.07.006 23968813

[ref44] Traub RJ, Inpankaew T, Sutthikornchai C, Sukthana Y and Thompson RC (2008) PCR-based coprodiagnostic tools reveal dogs as reservoirs of zoonotic ancylostomiasis caused by *Ancylostoma ceylanicum* in temple communities in Bangkok. *Veterinary Parasitology* 155(1-2), 67–73. 10.1016/j.vetpar.2008.05.001 18556131

[ref45] Tuli MD, Li H, Li S, Zhai J, Wu Y, Huang W, Feng Y, Chen W and Yuan D (2022) Molecular detection of a novel *Ancylostoma* sp. by whole mtDNA sequence from pangolin *Manis javanica*. *Parasites and Vectors* 15(1), 70. 10.1186/s13071-022-05191-0 35236404 PMC8889679

[ref46] Venkatesan A, Jimenez Castro PD, Morosetti A, Horvath H, Chen R, Redman E, Dunn K, Collins JB, Fraser JS, Andersen EC, Kaplan RM and Gilleard JS (2023) Molecular evidence of widespread benzimidazole drug resistance in *Ancylostoma caninum* from domestic dogs throughout the USA and discovery of a novel β-tubulin benzimidazole resistance mutation. *PLOS Pathogens.* 19(3), e1011146. 10.1371/journal.ppat.1011146 36862759 PMC10013918

[ref47] Walker M and Jacobs D (1985) Pathophysiology of *Uncinaria stenocephala* infections of dogs. *Veterinary Annual* 25, 263–271.

[ref48] Watkins CV and Harvey LA (1942) On the parasites of silver foxes on some farms in the south-west. *Parasitology* 34(2), 155–179. 10.1017/S0031182000016139

[ref49] Wolfgang RW (1956) *Dochmoides yukonensis* sp. nov. from the brown bear (*Ursus americanus*) in the Yukon. *Canadian Journal of Zoology* 34(1), 21–27. 10.1139/z56-003

[ref50] Xhaxhiu D, Kusi I, Rapti D, Kondi E, Postoli R, Rinaldi L, Dimitrova ZM, Visser M, Knaus M and Rehbein S (2011) Principal intestinal parasites of dogs in Tirana, Albania. *Parasitology Research* 108(2), 341–353. 10.1007/s00436-010-2067-8 20878182

[ref51] Xie Y, Xu Z, Zheng Y, Li Y, Liu Y, Wang L, Zhou X, Zuo Z, Gu X and Yang G (2019) The mitochondrial genome of the dog hookworm *Ancylostoma caninum* (Nematoda, Ancylostomatidae) from Southwest China. *Mitochondrial DNA Part B* 4(2), 3002–3004. 10.1080/23802359.2019.1666048 33365829 PMC7706842

